# Engineered Sumoylation-Deficient Prdx6 Mutant Protein-Loaded Nanoparticles Provide Increased Cellular Defense and Prevent Lens Opacity

**DOI:** 10.3390/antiox10081245

**Published:** 2021-08-04

**Authors:** Bhavana Chhunchha, Eri Kubo, Uday B. Kompella, Dhirendra P. Singh

**Affiliations:** 1 Ophthalmology and Visual Sciences, University of Nebraska Medical Center, Omaha, NE 68198, USA; 2 Department of Ophthalmology, Kanazawa Medical University, Kanazawa 9200265, Ishikawa, Japan; kuboe@kanazawa-med.ac.jp; 3 Departments of Pharmaceutical Sciences, Ophthalmology, and Bioengineering, University of Colorado Anschutz Medical Campus, Aurora, CO 80045, USA; Uday.Kompella@ucdenver.edu

**Keywords:** peroxiredoxin 6, transduction domain, oxidative stress, antioxidants, nano-formulation, nanoparticles, sumoylation, protective mutation

## Abstract

Aberrant Sumoylation-mediated protein dysfunction is involved in a variety of oxidative and aging pathologies. We previously reported that Sumoylation-deficient Prdx6K^(lysine)122/142R(Arginine)^ linked to the TAT-transduction domain gained stability and protective efficacy. In the present study, we formulated wild-type TAT-HA-Prdx6*^WT^* and Sumoylation-deficient Prdx6-loaded poly-lactic-co-glycolic acid (PLGA) nanoparticles (NPs) to further enhance stability, protective activities, and sustained delivery. We found that in vitro and subconjuctival delivery of Sumoylation-deficient Prdx6-NPs provided a greater protection of lens epithelial cells (LECs) derived from human and *Prdx6^−/−^*-deficient mouse lenses against oxidative stress, and it also delayed the lens opacity in Shumiya cataract rats (SCRs) than TAT-HA-Prdx6*^WT^*-NPs. The encapsulation efficiencies of TAT-HA-Prdx6-NPs were ≈56%–62%. Dynamic light scattering (DLS) and atomic force microscopy (AFM) analyses showed that the NPs were spherical, with a size of 50–250 nm and a negative zeta potential (≈23 mV). TAT-HA-Prdx6 analog-NPs released bioactive TAT-HA-Prdx6 (6%–7%) within 24 h. Sumoylation-deficient TAT-HA-Prdx6-NPs provided 35% more protection by reducing the oxidative load of LECs exposed to H_2_O_2_ compared to TAT-HA-Prdx6*^WT^*-NPs. A subconjuctival delivery of TAT-HA-Prdx6 analog-NPs demonstrated that released TAT-HA-Prdx6*^K122/142R^* could reduce lens opacity by ≈60% in SCRs. Collectively, our results demonstrate for the first time that the subconjuctival delivery of Sumoylation-deficient Prdx6-NPs is efficiently cytoprotective and provide a proof of concept for potential use to delay cataract and oxidative-related pathobiology in general.

## 1. Introduction

With advancing age or upon oxidative stress, the loss of genetically determined protective functions of antioxidant molecules leads to aging disorders, including blinding diseases. In young healthy cells, the antioxidant systems are well regulated and efficiently protect cells against various stressors, but with advancing age, the responses are debilitated, which results in enhanced susceptibility to oxidative stress-induced pathologies [1,2,3,4,5,6,7]. Various biological and chemical factors in the cellular and external environments, such as ultraviolet (UV) radiation, H_2_O_2_, and inflammatory cytokines have been demonstrated to generate reactive oxygen species (ROS), which further results in excessive oxidative stress-driven injurious signaling. To cope with this, the cells have evolved a variety of antioxidant genes, such as catalase (Cat), glutathione-peroxidase (Gpx), hemeoxygenase 1 (HO1), and peroxiredoxns (Prdxs). One of the antioxidant genes encodes peroxiredoxin 6 (Prdx6). The Prdxs family has six members of nonselenium enzymes that catalyze the reduction of hydrogen peroxide and organic peroxides [8,9,10,11,12,13,14,15,16,17]. Prdx1–5 contain two cysteine residues [18,19,20]. While unlike other members of the Prdx family, Prdx6 contains a single redox-active cysteine 47 (C47) lacking resolving cysteine [16,21,22].

Prdx6 is highly expressed in eye, brain, and lungs [3,9,12,15,19,23]. Prdx6 is a multifunctional cytoprotective protein with acidic calcium (Ca^2+^)-independent phospholipase A_2_ (aiPLA_2_), glutathione (GSH) peroxidase, and recently identified lysophosphatidylcholine acyl transferase (LPCAT) activities [10,24,25,26]. Prdx6 is predominantly present in the cytoplasm; importantly, it is also localized in ROS-generating organelles, such as mitochondria, cerebral fluid, endoplasmic reticulum, lysosome, plasma membrane, and lamellar body, suggesting its important biological functions in maintaining redox homeostasis [16,27]. We and other investigators have shown that Prdx6 expression is essential to control cellular homeostasis, and it does so by regulating the Ca^2+^ and the intracellular redox homeostasis [3,8,9,11,12,16,17,28,29,30,31,32]. The overexpression of Prdx6 in different cell types has been found to protect from ROS-induced cellular injuries [9,11,16,28,33,34,35]. Conversely, a deficiency of Prdx6 results in the failure of cellular homeostasis and cell death [9,11,14,33,36,37,38]. Furthermore, Prdx6-deficient mice has been found to be highly sensitive to oxidative stress-induced damage [3,36]. Previously, we reported that the subconjunctival delivery of Prdx6-linked to TAT (Transcriptional Activator of Transcription) transduction domain protected lens cells and delayed cataract formation in Shumiya cataract rats [14]. These studies suggest that Prdx6 is a critical biomolecule to maintain cells/tissues/organs health.

Moreover, the maintenance of protein homeostasis is essential to maintain cellular systems during the progressive increase of oxidative load during aging [9,39,40]. The loss of protein homeostasis has been found to be related to oxidative stress-induced aberrant post-translational modifications [9,41]. Post-translational modifications, such as Sumoylation, phosphorylation, acetylation, and glycosylation play a diversified role(s) in the regulation of protein’s functions [9,10,39,42,43]. Aging- or oxidative stress-evoked aberrant Sumoylation of proteins has been demonstrated in the initiation and development of various types of human disorders [39,44,45,46]. Sumos (Small ubiquitin-like modifiers) are important post-translational modifiers and thereby involved in the regulation of various cellular processes by affecting protein integrity and activities. Mostly, transcription factors are targets for Sumoylation, but several non-nuclear/cytoplasmic proteins, including Prdx6 have been reported to be Sumoylated [9,10,47]. Sumoylation occurs predominately at a consensus motif present in substrates (Ψ-K-X-[D/E], where Ψ is any large hydrophobic amino acid (I, V, or L), K is the target lysine, X is any residue, and D/E is aspartate or glutamate) [48,49]. However, an extended site(s) for Sumo binding has been identified and is termed as a non-consensus site for Sumos conjugation [9,50,51]. We have shown that Prdx6 is aberrantly Sumoylated by Sumo1 in response to oxidative stress and aging, resulting in the loss of its function and stability [9,10]. We also have identified lysine (K) 122 and K142 as the major Sumo1 binding sites in Prdx6 protein, and the Sumoylation-deficient Prdx6*^K122/142R^* gained the stability and enzymatic activities with increased cytoprotection [10,11,14].

Recent evolution in genes or proteins delivery coupled with the identification of several protein transduction domains (PTDs), such as human immunodeficiency virus transcriptional activator of transcription (TAT) PTD (YGRKKRRQRRR) has made it possible to deliver genes or its products to cells or organs in vitro and in vivo [14,52,53,54,55,56]. A wealth of literature, including our own studies, have reported that PTD-linked proteins can internalize into cells or organs in a concentration-dependent fashion [53,57,58,59]. In addition, the development of nanoformulations of drug/protein carrier systems has been found to be highly successful, and they have become a promising aspect in the field of drug delivery. Polymer-based nano- and microparticles loaded with drugs serve as a reservoir capable of sustaining drugs or biomolecules release for a prolonged time by maintaining their stability and activities [60,61,62]. It has been reported that they remain in the subconjunctival space for prolonged periods to sustain drug release [63,64]. Thus, in this work, to postpone or treat cataracts, which is one of the huge problems of blinding disorders, we intended to enhance further the stability and activity of WT-Prdx6 or Sumoylation-deficient Prdx6. PLGA-based nanoformulation has been successfully developed, and it has been found to be safe and biocompatible with polymers for the therapeutic treatment of various diseases, including ocular disease [65,66,67,68,69,70]. Backed with these facts, we hypothesized that protein transduction domain (TAT)-fused Prdx6 protein-loaded PLGA-NPs should have a better protective potential than necked TAT-HA-Prdx6 protein delivery [14] when delivered in vitro or in vivo. Hence, in this study, we prepared wild-type TAT-HA-Prdx6*^WT^* or its mutant, Sumoylation-deficient Prdx6*^K122/142R^* at its redox-active cysteine 47 (C47) and catalytic traid in the PLA_2_ site, Serine (S) 32-Histidine (H) 26-Aspartic acid (D) 140 (inactive-mutant)–loaded PLGA-NPs for the sustained release of Prdx6 analog and examined their cytocompatibility, sustainability, and protective efficacy in vitro and in vivo. Using lens epithelial cells derived from lenses of human, *Prdx6*-deficient mice, and SCRs (a model for cataract) as model systems, we found a sustained release of encapsulated TAT-HA-Prdx6 analog, and the released TAT-HA-Prdx6 or its mutant at Sumoylation sites was biologically active and retained Prdx6 integrity. Fascinatingly, LECs treated with Sumoylation-deficient Prdx6*^K122/142R^*-NPs displayed a significantly higher resistance, with reduction in oxidative load facing H_2_O_2_-induced stress compared to TAT-HA-Prdx6*^WT^*-NPs. Intriguingly, the subconjunctival delivery of analog of TAT-HA-Prdx6-NPs in SCR showed that TAT-HA-Prdx6-NPs released TAT-HA-Prdx6 analog in vivo, and TAT-HA-Prdx6*^K122/142R^*-NPs could be more efficient in reducing lens opacity and delay cataract formation. Thus, our findings demonstrate that TAT-linked Sumoylation-deficient Prdx6 in the form of TAT-HA-Prdx6-NPs can have a greater potential to block oxidative stress and its induced aberrant Sumoylation signaling-mediated etiopathology.

## 2. Materials and Methods

### 2.1. Animals

Animal experiments were conducted according to the U.S. National Institute of Health Guide for the Care and Use of Laboratory Animals, the recommendation and statement of ARVO for the Use of Animals in Ophthalmic and Vision Research, and the Guidelines for the use of Laboratory Animals Kanazawa Medical University and University of Nebraska Medical Center (UNMC), Omaha NE. All experimental procedures on animals were approved by the Institutional Animal Care and Use Committee (IACUC), UNMC (IACUC no. 18-058-05-FC) and the committee of Animal Research at Kanazawa medical University, Kanazawa, Japan (Permission no. 2017-07). The rats or mice used were housed in pathogen-free facilities (12 h light–dark cycle) and fed a diet of regular chow. In this work, we used 6-week-old SCR rats to inject TAT-HA-Prdx6 analog-NPs in the SCR eyes. These animals develop posterior cortical cataracts at 9 weeks old and mature or severe cataracts after 10 week onwards (S. Shumiya, 1995) [71]. Before treatment, four-week-old cataractous (Cat+) and noncataractous (Cat-) SCRs were identified via performing genomic polymerase chain reaction (PCR) with genomic DNA from the rat’s tails and primers specific to lanosterol synthetase (Lss) [72]. The yielded product was resolved onto 15% polyacrylamide gel electrophoresis (PAGE) or onto 2.5% agarose gel electrophoresis to detect the mutation of lanosterol synthetase (Lss) as described previously [72]. SCRs showing positive Lss mutation were used for the study. A total of twelve SCR were used to examine the protective effect of TAT-HA-Prdx6 analog-NPs. The SCRs (6-week-old) were divided in two groups (*n* = 6 (12 eye/lenses) in each group) for the administration of TAT-HA-Prdx6 analog-NPs, as described in figure legend of Figure 6. The right eye of each group of SCR received TAT-HA-Prdx6*^WT^*-NPs or Sumoylation-deficient TAT-HA-Prdx6*^K122/142R^*-NPs, while the left eyes received TAT-HA-Prdx6*^Inactive-mutant^*-NPs suspended in physiological saline as a vehicle control. Then, 10 µL NPs containing 25 µg of protein of each preparation suspended in physiological saline was used for subconjunctival administration to each eye. Notably, our aim was to use the minimum number of animals that could give statistically significant results. After the end of the experiments, SCRs were humanely sacrificed using CO_2_, and lenses were carefully isolated. Isolated lenses from each group were photographed by a stereoscopic microscope with dark-field illumination (SMZ745T, Nikon). Level of lens opacity was analyzed in each digital photograph of rat lenses by using MultiGauze Software (Fujifilm, Tokyo, Japan).

### 2.2. Human Lens Epithelial Cells

Human Lens Epithelial Cells (hLECs) (a kind gift of the late Dr. Venkat N. Reddy, Eye Research Institute, Oakland University, Rochester, MI, USA) were maintained by culturing in DMEM (Dulbecco’s modified Eagle’s medium; Invitrogen, Carlsbad, CA, USA) with 15 to 20% FBS (fetal bovine serum; Atlanta Biologicals, Inc., Flowery Branch, GA, USA), 100 µg/mL streptomycin, and 100 µg/mL penicillin in a 5% CO_2_ environment at 37 °C as described previously [9].

### 2.3. Generation and Validation of Mouse LECs Isolated from Lenses of Prdx6^−/−^ and Prdx6^+/+^ Mice

Studies were approved by the University of Nebraska Medical Center, Omaha, NE, USA. LECs isolated from Prdx6 knockout (*Prdx6*^−/−^) and wild-type (*Prdx6*^+/+^) mice were generated and maintained routinely in Dulbecco’s Modified Eagle’s Medium (DMEM; Invitrogen, Carlsbad, CA, USA) containing 10% FBS (Atlanta Biologicals, Inc., Flowery Branch, GA, USA) as described earlier [3]. For the studies, we used *Prdx6^−/−^* mice, which are maintained on fully inbred C57BL/6 background, and wild-type mice of the same sex and age (*Prdx6^+/+^*). This minimizes the changes or variation due to genetic background. All mice were maintained under specific pathogen-free conditions in an animal facility. LECs were isolated from mice of identical age, and Western analysis was performed to validate the presence of αA-crystalline [3], which is a specific marker of LECs.

### 2.4. Expression and Purification of Recombinant Protein, TAT-HA-Prdx6

A human LEC cDNA library was used to isolate a full-length of Prdx6 cDNA by using Prdx6-specific Forward primer (5′-GTCGCCATGGCCGGAGGTCTGCTTC-3′ containing *NcoI* site) and Reverse primer (5′-AATTGGCAGCTGACATCCTCTGGCTC-3′) [73]. The resultant amplified PCR products were purified from agarose gel electrophoresis (QIAEX II Gel Extraction Kit, Cat No. 20021, Qiagen Inc., Valencia, CA, USA). The purified DNA was cloned into a TA-cloning vector (Invitrogen, Carlsbad, CA, USA) and then transformed into competent cells. Plasmids were isolated and purified from selected single colonies. A plasmid consisting of Prdx6 cDNA was amplified and cloned into a pTAT-HA vector at *NcoI* and *EcoRI* sites (a kind gift of Dr. S. F. Dowdy) [14,28,73]. Then, wild-type TAT-HA-Prdx6 was mutated at K (lysine) 122/142R (arginine), Cysteine (C) 47 IL (Isoleucine) and Serine (S) 32-Histidine (H) 26-Aspartic acid (D)140 to Alanine by using a Site-Directed Mutagenesis (SDM) kit as mentioned below (Section 2.5). TAT-HA-Prdx6*^WT^* and its mutants at K122/142R and inactive–mutant (C47IL/S32A/H26A/D140A) plasmids were transformed into Escherichia coli BL21 (DE3). The transformants were selected on a Luria broth (LB) plate containing 100 µg/mL ampicillin. The selected single colonies were cultured in 10 mL of LB medium containing ampicillin at 37 °C with continuous shaking at 200 rpm overnight. The next day, 10 mL of overnight culture was added into 250 mL of prewarm LB–Ampicillin medium with vigorous shaking at 37 °C until an OD_600_ reached 0.6. Then, an 0.5 mL sample was taken out and kept for expression control. Thereafter, protein expression of the transformants was induced by adding 1 mM IPTG in the flask, and then further along, the culture was incubated at 37 °C with vigorous shaking. After 5 h, 0.5 mL of sample was taken out for expression analysis. The samples were centrifuged, and pellets of the cells obtained from 0.5 mL collected samples before and after IPTG induction were used for SDS-PAGE analysis for Prdx6 expression, as reported earlier [9,10,14,28]. Finally, recombinant Prdx6 protein was purified after thawing the pellet for 15 min on ice using QIAexpress^®^ Ni-NTA Fast Start kit column (Qiagen Inc., Valencia, CA, USA) as described previously [4,9]. This purified Prdx6 protein was quantified and kept at −80 °C until use.

### 2.5. Site-Directed Mutagenesis (SDM)

PCR-based site-directed mutagenesis was performed using the QuikChange™ site-directed mutagenesis kit (Invitrogen, Carlsbad, CA, USA), following the company’s protocol and our published reports [9,10,11]. Primers used for SDM were as follows.

➢ Prdx6 K 122 to R122, (K122R)

Forward primer: 5′-GGCATGCTGGATCCAGCAGAGAGGGATGAAAAGGGC-3′

Reverse primer: 5′-GCCCTTTTCATCCCTCTCTGCTGGATCCAGCATGCC-3′

➢ Prdx6 K142 to R142, (K142R)

Forward primer: 5′-GGTCCTGATAAGCGGCTGAAGCTGTCTATCCTCTACCC-3′

Reverse primer: 5′-GGGTAGAGGATAGACAGCTTCAGCCGTTATCAGGACC-3′

➢ Prdx6 Cysteine (C) 47 to isoleucine (IL) 47, (C47IL)

Forward primer: 5′-CTTTACCCCAGTGATAACCACAGAGGTTGGCAGAGC-3′

Reverse primer: 5′-GCTCTGCCAAGCTCTGTGGTTATCACTGGGGTAAAG-3′

➢ Prdx6 Histidine (H) 26 to Alanine (A) 26, (H26A)

Forward primer: 5′-CGGCCGCATCCGTTTCGCCGACTTTCTGGGAGACTC-3′

Reverse primer: 5′-GAGTCTCCCAGAAAGTCGGCGAAACGGATGCGGCCG-3′

➢ Prdx6 Serine (S) 32 to A32, (S32A)

Forward primer: 5′-CCACGACTTTCTGGGAGACGCATGGGGCATTCTCTTCTCC-3′

Reverse primer: 5′-GGAGAAGAGAATGCCCCATGCGTCTCCCAGAAAGTCGTGG-3′

➢ Prdx6 Aspartic Acid (D) 140 to A140, (D140A)

Forward primer: 5′-GTGGTGTTTGTTTTTGGTCCTGCAAAGAAGCTGAAGCTGTCTATCC-3′

Reverse primer: 3′-GGATAGACAGCTTCAGCTTCTTTGCAGGACCAAAAACAAACACCAC-3′

### 2.6. Coomassies Blue Staining and Western Blot Analysis

Wild-type (WT) TAT-HA-Prdx6 and its Sumoylation-deficient, at Lysine (K) 122/142 to arginine (R) and inactive-mutant (Cysteine (C) 47 to Isoleucine (IL)/Serine(S) 32 to Alanine (A) 32/Histidine (H) 26 to A26/Aspartic Acid (D) 140 to A140) recombinant protein samples were collected before and after IPTG treatment of E. coli BL21 (DE3) bacteria containing cDNA of the above-mentioned molecules (under the Section 2.4, ‘Expression and purification of recombinant protein, TAT-HA-Prdx6’). An equal amount of bacterial lysate or purified protein isolated from the lysate(s) were resolved onto 10% or 4%–20% SDS-PAGE. The gel containing resolved protein bands was subjected to Coomassie blue staining for photography or immunoblotted onto a PVDF membrane (Perkin Elmer, Waltham, MA, USA) using antibody specific to Prdx6 (Ab Frontier, Seoul, Korea), visualized, and the images were recorded with a Fujifilm-LAS-4000 luminescent image analyzer (Fujifilm Medical Systems Inc., Hanover Park, IL, USA), as described previously [9,10].

### 2.7. Preparation of Nanoparticles Containing TAT-HA-Prdx6 Analog

TAT-HA-Prdx6 protein analog loaded nanoparticles (NPs) were nanoformulated by using a water-in-oil-in-water (*w/o/w*) emulsion solvent evaporation technique, as described previously [61,74] with a modified composition designed to facilitate the release of the encapsulated protein in its active form. Briefly, 100 mg (+/−5 mg) PLGA (Poly (D, L-lactic-co-glycolic acid) with a lactide/glycolide ratio of 50:50 and Inherent Viscosity: 0.55–0.75 dL/g (Lactel Absorbable Polymers; Catalog No. B6010-2)) was dissolved in 4 mL of dichloromethane (DCM; Catalog No. 270997, Sigma Aldrich, St. Louis, MO, USA) along with or without 5 µg of Coumarin 6 (a florescence tracking dye, stock solution 0.2 mg/mL in DCM; Catalog No. 442631, Sigma Aldrich) to form a polymer solution. To obtain the primary emulsion with a protein-to-polymer ratio of 1:10; an aqueous solution consisting of 10 mg of TAT-HA-Prdx6-analog protein along with 15 mg of rat serum albumin (RSA) in purified water was emulsified into the polymer solution by vortexing for 1–2 min. The primary emulsion was added dropwise into a ≈ 35 mL of 0.3% Vitamin E-TPGS (D-α-Tocopherol polyethylene glycol 1000 succinate; Catalog no. 57668, Sigma Aldrich) with continuous vortexing followed by sonication for 20 min (5-5-5-5 min continuous sonication with interval of 5 min) on an ice bath at 40% Amplitude and ≈18 W of energy output (Misonix S-4000, Ultrasonic Liquid Processor Sonicator, Farmingdale, NY, USA). This multiple emulsion was stirred overnight on a magnetic stir plate at room temperature for the evaporation of DCM in the dark. Finally, the NPs were collected by ultracentrifugation at 30,000 rpm for 20 min at 4 °C (Optima XE-90 Ultracentrifuge, Beckman Coulter, Indianapolis, IN, USA). The NPs were washed three to four times with water to remove Vitamin E-TPGS and free proteins. The supernatant and washing solution from NPs preparations were collected, and the amount of protein that was not encapsulated was assessed. From the total amount of protein added and the amount not encapsulated, the protein loading in the PLGA-NPs was determined. The incorporation of RSA into the formulation process was used to stabilize the TAT-HA-Prdx6 analog from interfacial inactivation and to facilitate the release of the TAT-HA-Prdx6 analog from the NPs. After the final centrifugation, the nanoparticles were resuspended in 5 mL of distilled water and sonicated as described above for 30 s on an ice bath. To remove aggregates, the NPs were centrifuged at 1000 rpm for 10 min at 4 °C. The supernatant was collected, and some desired amounts of NPs were used for their characterization. The remaining NPs suspension was stored at −80 °C for 24 h and subsequently lyophilized (Freeze Dryer, LABCONCO, Kansas City, MO, USA) for further use in the experiments.

### 2.8. Characterization of Size and Zeta Potential of Nanoparticles

The hydrodynamic diameter of NPs was measured before and after lyophilization; the particle size before the lyophilization of nanoparticles was measured by diluting in distilled water. To measure the diameter of nanoparticles after lyophilization, a suspension of NPs at 0.5 mg/mL of distilled water was prepared by sonication for 1–2 min using a microtip probe sonicator. The intensity-mean Z-averaged hydrodynamic diameter (Deff) and zeta (ζ)-potential of particles were determined using dynamic light scattering (DLS) and a Zetasizer NanoZS (Marvern Instrument Ltd., Malvern, UK). All measurements were performed in automatic mode at 25 °C. Software provided by the manufacture was used to calculate the size, polydispersity indices (PDIs), and ζ-potential of nanoparticles. All measurements were performed at least in triplicate to calculate the mean values ± SD.

### 2.9. Atomic Force Microscopy (AFM) Imaging and Image Analysis of Nanoparticles

AFM imaging and image analysis of TAT-HA-Prdx6 analog-loaded NPs were performed at the Nanoimaging core facility of the University of Nebraska Medical Center, Omaha, NE, USA. In brief, freshly cleaved mica was altered with the treatment of APS (1-(3-aminopropyl) silatrane) as described previously [75,76]. The TAT-HA-Prdx6 analog-NPs samples were diluted in water and deposited onto APS treated mica for 2 min. The samples were rinsed with deionized water and dried with a gentle flow of argon. The image was recorded with the Multimode Nanoscope IV system (Bruker Instruments, Santa Barbara, CA, USA) in Tapping Mode at ambient conditions. Silicon probes RTESPA-300 (Bruker Nano Inc., CA, USA) with a resonance frequency of ≈300 kHz and a spring constant of ≈40 N/m were applied for imaging at scanning rate of 0.5–1.0 Hz. Images were processed using the FemtoScan software package (Advanced Technologies Center, Moscow, Russia). The size of nanoparticles was measured automatically with Enum features or manually with the Cross-section tool in the FemtoScan program.

### 2.10. Quantification of Protein Encapsulation Efficiency (EE)

Protein encapsulation efficiency (EE) were determined in accordance with previously published methods [77,78,79]. Briefly, the amount of TAT-HA-Prdx6 analog plus RSA protein absorbed in the NPs was assessed by subtracting the amount of TAT-HA-Prdx6 analog plus RSA protein, present in the combined supernatants removed after centrifugation during the nanoparticle preparations and washes, from the initial quantity of TAT-HA-Prdx6 analog plus RSA protein used for nanoparticle preparation. The protein content was measured using Pierce^TM^ BCA protein analysis (Catalog No. 23225, ThermoFisher Scientific, Waltham, MA, USA) and a calibration curve. The EE was determined as follows:

$$\text{Encapsulation efficiency (\%) = }\frac{\mathrm{Initial}\;\mathrm{protein}\;\mathrm{concentration}-\mathrm{unencapsulated}\;\mathrm{protein}\;\mathrm{concentration}}{\mathrm{Initial}\;\mathrm{protein}\;\mathrm{concentration}\;}\times \;100.$$

### 2.11. Prdx6 Analog Release Assay from Nanoparticles In Vitro

To examine the TAT-HA-Prdx6 analog release pattern from nanoparticles, we first determined a suitable buffer to avoid the degradation of TAT-HA-Prdx6 analog under the conditions used for the release assay. Toward this, we chose phosphate-buffered saline (PBS, pH 7.3) with 0.025% sodium azide (as a preservative) for the in vitro release medium, as reported earlier by several investigators [80,81]. Three mg of lyophilized NPs were suspended in 3 mL of PBS by vortexing, and then, the suspension was equally divided into 1.5 mL aliquots in Eppendorf tubes. The tubes containing suspension were incubated under the condition used for the release study, i.e., at 37 °C on a shaker rotating at 300 rpm (Innova^TM^ 4000 Incubator Shaker, New Brunswick Scientific, Midland, ON, Canada). At predetermined time intervals, the tubes were taken out, and the samples were centrifuged. Protein concentration was measured from all collected and remaining released protein samples, and they were stored at −80 °C. These samples were used to quantify Prdx6 analog protein with sandwich ELISA as well as to measure Prdx6 activities as described below. The amount of protein in the samples was determined using a BCA protein assay kit (ThermoFisher Scientific, USA).

### 2.12. Sandwich-ELISA (Enzyme Linked Immunosorbent Assay)

To assess in vitro released TAT-HA-Prdx6 protein analog (Figure 3A,D), we have performed Sandwich-ELISA (Abnova, Taipei City, Taiwan) as described previously [9]. In vitro released protein was quantified by a BCA protein assay kit, and an equal amount of protein was loaded into an ELISA plate-well precoated with anti-Prdx6 polyclonal antibody (LS-C404242, LifeSpan BioSciences, Inc, Seattle, WA, USA) followed by incubation with monoclonal anti-Prdx6 antibody (LF-MA0018, Ab Frontier, South Korea). After incubation with mIgGk BP-HRP conjugated secondary antibody (sc-516102, Santa Cruz Biotechnology, Dallas, TX, USA), OPD substrate was added for color development, and O.D. was recorded at 450 nm.

### 2.13. Measurement of Phospholipase A_2_ (PLA_2_) Activity

To determine the Prdx6 PLA_2_ activity of in vitro released TAT-HA-Prdx6 protein analog from NPs as well as TAT-HA-Prdx6 analog-NPs-treated mLECs or subconjuctivally injected rat lenses in vivo, we applied Phospholipase A_2_ (PLA_2_) activity assay following the company’s instructions (EnzChek Phospholipase A2 kit; E10217, Invitrogen) and our previously published protocol [9,10]. Briefly, stock solution of PLA_2_ (500 Units/mL) was diluted with 1× reaction buffer to prepare different concentrations (0–10 units/mL) of PLA_2_ to plot a standard curve. For the sample, in vitro released protein from TAT-HA-Prdx6 analog-NPs or the total protein from TAT-HA-Prdx6 analog-NPs treated mLECs or lenses isolated from subconjuctivally injected SCR with the NPs were quantified by BCA protein assay. There was an equal amount of protein by volume (from in vitro release, Figure 3B) or equal amount of protein by concentrations (from LECs treated with the NPs, Figure 5E and SCR lenses administered with forementioned NPs, Figure 6C), and all samples were diluted with 1× PLA2 reaction buffer up to 50 µL volume. Thereafter, 50 µL of the substrate–liposome mix was added to each microplate well containing standards, samples, and controls to start the reaction with a total volume of 100 µL. Finally, the intensity of fluorescence of each well was measured at Ex485 nm/Em535 nm using a microplate reader (DTX 880, Multimode Detector, and Molecular Device) and presented.

### 2.14. Glutathione (GSH) Peroxidase Activity

To examine the Prdx6 GSH peroxidase activity of in vitro released TAT-HA-Prdx6 protein analog (Figure 3C,F) from NPs as well as Prdx6 protein from TAT-HA-Prdx6 analog-NPs treated LECs and SCR rat lenses (Figures 5F and 6D), we performed GPx activity assay in accordance with the company’s protocol (GSH Peroxidase activity kit, Cat No. ADI-900-158, Enzo Life Sciences) and our published reports [9,10]. In brief, in vitro released protein and protein isolated from LECs or SCR eye lenses were quantified and equalized by volume (Figure 3C,F) and concentrations (Figures 5F and 6D), respectively. Then, 140 µL of 1× assay buffer, 20 µL of 10× reaction buffer, and 20 µL of glutathione peroxidase, controls, and samples were added to a 96 well-plate. Thereafter, the reaction was initiated by adding 20 µL of cumene hydroperoxide to each well. O.D. was recorded at 340 nm every 1 min over a 10–15-min period. The O.D. of the blank is subtracted from the O.D. of the standard as well as the sample to obtain the net rate of absorbance at 340 nm for the calculation of GSH activity in the samples [9,10].

### 2.15. Cellular Uptake of Prdx6 Protein Analog-Loaded Nanoparticles

The cellular uptake of Prdx6 analog-NPs was assessed by florescence microscopy (Nikon microscope, Nikon Instruments Inc, Melville, NY, USA). hLECs (1 × 10^6^), *Prdx6^+/+^* (1.5 × 10^6^) and *Prdx6^−/−^* (1.5 × 10^6^) were cultured in 100 mm culture dishes. Then, 24 h later, the growth medium was removed, and the cells monolayer was washed with sterile phosphate-buffered saline (PBS). A stock suspension of Prdx6 analog-loaded PLGA nanoparticles (10 µg/mL protein) was resuspended by vortexing in complete growth medium, and the medium in the plates was replaced with the suspension of TAT-HA-Prdx6 analog-NPs and incubated for 6 h. Cells were trypsinized and seeded in 96-well plates with a glass coverslip and 100 mm culture petri dishes for ROS and MTS assays, photomicrography, and protein isolation, respectively. Coumarin-6, a fluorescence dye, was used to locate the green fluorescence for cellular uptake of NPs. From the fluorescence image, we can observe a green florescence in the cell cytoplasm, which reflects the penetration of TAT-HA-Prdx6 analog-loaded NPs.

### 2.16. Fluorescence Image and DAPI Staining

hLECs or mouse *Prdx6^+/+^* or *Prdx6^−/−^* LECs were incubated with TAT-HA-Prdx6 analog-NPs suspension. After 6 h, LECs were trypsinized and harvested onto the coverslip. After 24 h of cells harvesting, coverslips containing cells were washed three times with 1X PBS. Cells were fixed in freshly prepared 3.7% formaldehyde for 10 min. Cells were washed three times with PBS to remove fixative, and then cells were permeabilized with 0.1% Triton X-100 (prepared in PBS) briefly. After washing with PBS, 10 µL of DAPI (1 mg/mL stock) diluted in 10 mL of PBS was added to coverslips containing cells and incubated in dark for 10 min at room temperature. Finally, coverslips containing cells were washed three times and mounted. Images were taken using different filter settings, such as Phase, FITC (Coumarin 6), and DAPI with Nikon fluorescence microscope.

### 2.17. Quantitation of Intracellular ROS Level by H2-DCF-DA and CellROX^®^ Deep Red Reagent

Intracellular ROS were quantified by using fluorescent dye dichlorofluorescin diacetate (H2-DCF-DA), which is a nonpolar compound that is converted into a polar derivative (dichlorofluorescein) by cellular esterase after incorporation into cells [4,8,39,82]. *Prdx6^+/+^* and *Prdx6^−/−^* mLECs were incubated with TAT-HA-Prdx6 analog-NPs suspension. Then, 6 h later, LECs were trypsinized and seeded into 96-well plates. After 24 h, cells were submitted H_2_O_2_ exposure as indicated in the figure legends. After 8 h of H_2_O_2_ exposure, the medium was replaced with Hank’s solution containing 10 µM H2-DCF-DA dye, and cells were incubated. Then, 30 min later, a Spectra Max Gemini EM (Mol. Devices, Sunnyvale, CA, USA) was used to record intracellular fluorescence with excitation (Ex) at 485 nm and emission (Em) at 530 nm.

For the estimation of ROS levels, LECs were incubated with TAT-HA-Prdx6 analog-NPs suspension for 6 h. Thereafter, LECs were trypsinized and seeded in 96-well plates. Then, 24 h later, cells were exposed to H_2_O_2_, and after 8 h of H_2_O_2_ exposure, ROS levels were quantified following the company’s protocol (CellROX^®^ Deep Red Oxidative Stress Reagent, Catalog No. C10422). Briefly, a final concentration of 5 µM of CellROX deep red reagent was added to each well of a 96-well plate containing cells, and they were incubated at 37 °C for 30 min. Medium containing CellROX deep red reagent was aspirated from a 96-well plate, and cells were fixed with 3.7% formaldehyde. Then, 15 min later, the fluorescence signal was recorded at Ex640 nm/Em665 nm.

#### Determination of ROS Levels by H2-DCF-DA in SCR Eye Lenses Ex Vivo

ROS levels of SCR lenses were measured as described in our recent publication [83]. Briefly, SCRs were sacrificed by cervical dislocation, and lenses were isolated and were immediately frozen at −80 °C. The lenses were thawed on ice and homogenized (100 mg/mL) with freshly prepared buffer (50 mM phosphate buffer containing 1 mM EDTA, 0.5 mM PMSF, 1 µM Pepstatin, 80 mg/L Trypsin Inhibitor, pH 7.4). Then, 30 µM of H2-DCF-DA dye was added into a 96-well plate containing freshly prepared lens homogenate. After 30 min of incubation at 37 °C, fluorescence was recorded at excitation (Ex) at 485 nm and emission (Em) at 530 nm using a Spectra Max Gemini EM (Mol. Devices, Sunnyvale, CA, USA) [84,85,86].

### 2.18. Cell Viability Assay

Human or mouse (*Prdx6^+/+^* or *Prdx6^−/−^*) LECs were incubated with TAT-HA-Prdx6 analog-NPs suspension for 6 h. Thereafter, LECs were trypsinized and seeded in a 96-well plate. Then, 24 h later, cells were exposed with H_2_O_2_ to produce oxidative stress. The MTS assay of cell viability (Promega, Madison, WI, USA) uses 3-(4,5-dimethylthiazol-2-yl)-5-(3-carboxymethoxyphenyl)-2 to 4-sulphophenyl) 2H-tetrazolium salt [3,14]. When added to a medium having live cells, MTS is reduced to a water-soluble formazan salt. The *A*_490nm_ value (A = absorbance) was recorded after 2 to 4 h with a microplate reader. Values were normalized with absorbance of the respective control(s), and the results were presented.

### 2.19. Subconjuctival Delivery of Prdx6 Analog-NPs into SCR Ocular Lenses

To evaluate the potential of TAT-HA-Prdx6 analog-NPs in preventing the progression of cataract formation in SCR rats, we injected the TAT-HA-Prdx6 analog-NPs subconjunctivally. To minimize pain, before subconjuctival injection and another experimental procedure, we applied a drop of 0.5% proparacaine HCl to the eye. Six-week-old Shumiya cataract rats were used to perform experiments. The right eye of each rat was injected with TAT-HA-Prdx6*^WT^*-NPs or TAT-HA-Prdx6*^K122/142R^*-NPs, whereas the left eyes were administered with TAT-HA-Prdx6*^Inactive-mutant^*-NPs. The subconjuctival injection was made using micro-syringes fitted with 30-gauge needles. After 21 days, lenses were isolated from the rat eyes and photographed. At the end of the experiment, LECs were carefully detached/separated from the lenses, and extracts were prepared, and an equal amount of protein was used to measure PLA_2_ or GSH peroxidase activity, and LECs were used to quantify ROS levels.

### 2.20. Statistical Analyses

Data are presented as mean ± S.D. of the indicated number of experiments. For all data, statistical analysis was performed by Student’s *t*-test and/or one-way ANOVA when appropriate. A significant difference between the control and treatment group was determined as a *p* value of *, *p* < 0.050 and **, *p* < 0.001 for two or more independent experiments.

## 3. Results

### 3.1. A Schematic Diagram of Engineered TAT-HA-Prdx6 Expression Vector System and Expression Analysis of TAT-Linked Prdx6 Recombinant Proteins, TAT-HA-Prdx6^WT^ or TAT-HA-Prdx6^K122/142R^ or TAT-HA-Prdx6^Inactive-mutant^

**Figure 1:**
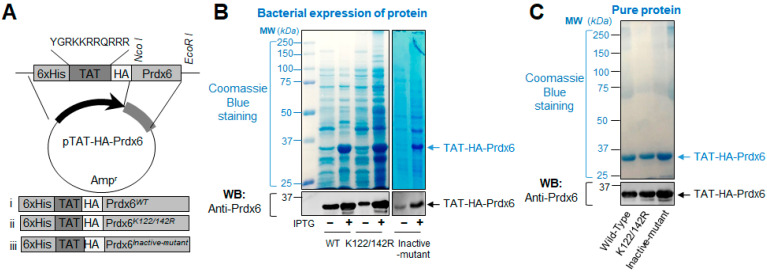
(**A**) An illustration of the TAT-HA-Prdx6 expression vector system. Constructs i, ii, and iii represent TAT-HA-Prdx6*^WT^*, Sumoylation-deficient TAT-HA-Prdx6*^K122/142R^*, and TAT-HA-Prdx6*^C47IL/S32A/H26A/D140A^* (inactive-mutant), respectively. These plasmids were utilized for recombinant protein preparation. (**B**,**C**) Expression of TAT-HA-Prdx6*^WT^*, and its mutant TAT-HA-Prdx6*^K122/142R^* and TAT-HA-Prdx6*^Inactive-mutant^* fusion recombinant protein in *Escherichia coli*. (**B**) TAT-HA-Prdx6*^WT^* or its mutant at K122/142R and inactive-mutant (C47IL/S32A/H26A/D140A) fusion bacterial protein expression was analyzed using Coomassie blue staining of SDS-PAGE gel (upper panel) and immunoblot analysis with anti-Prdx6 antibody (lower panel). (**C**) Purified TAT-HA-Prdx6*^WT^* and its mutants TAT-HA-Prdx6*^K122/142R^* and TAT-HA-Prdx6*^Inactive-mutant^* protein were analyzed using Coomassie blue staining (upper panel) and immunoblot analysis with Prdx6 antibody (lower panel).

Based on our previous studies showing that transduction domain, TAT-fused Prdx6 and its mutant, Sumoylation-deficient Prdx6 protein could internalize in lenses and lens cells in vitro and in vivo, and thereby, Sumoylation-deficient Prdx6 was more stable with higher enzymatic activities and provided a better protection in response to oxidative stress and aging [9,10,14]. Observing that the Sumoylation-deficient Prdx6 has better therapeutic potential, we intended to further enhance Prdx6 activity and its sustained delivery. To achieve this, we prepared and purified recombinant Prdx6 protein and its analog, TAT-HA-Prdx6 or TAT-HA-Prdx6*^K122/142R^* (mutated at Sumoylation sites) or TAT-HA-Prdx6*^C47IL/S32A/H26A/D140A^* (control, inactive-mutant) as described in Materials and Methods and Figure 1A as well as in our previously published reports [9,10,11]. We confirmed the integrity and activity of purified recombinant protein using immunoblot analysis and cell survival assay (MTS assay, data not shown). The assays revealed that all recombinant TAT-HA-Prdx6 analogs were abundantly expressed in the expression system and the expressed and purified proteins were biologically active (data not shown) and had ~35 kDa on SDS-PAGE as expected [9,10,14,28] (Figure 1B,C), demonstrating that the integrity of the recombinant TAT-HA-Prdx6 analog was well retained.

### 3.2. Encapsulation and Physical Characterization of TAT-Linked Prdx6 Recombinant Proteins, TAT-HA-Prdx6^WT^ or TAT-HA-Prdx6^K122/142R^ or TAT-HA-Prdx6^Inactive-mutant^-NPs

**Figure 2:**
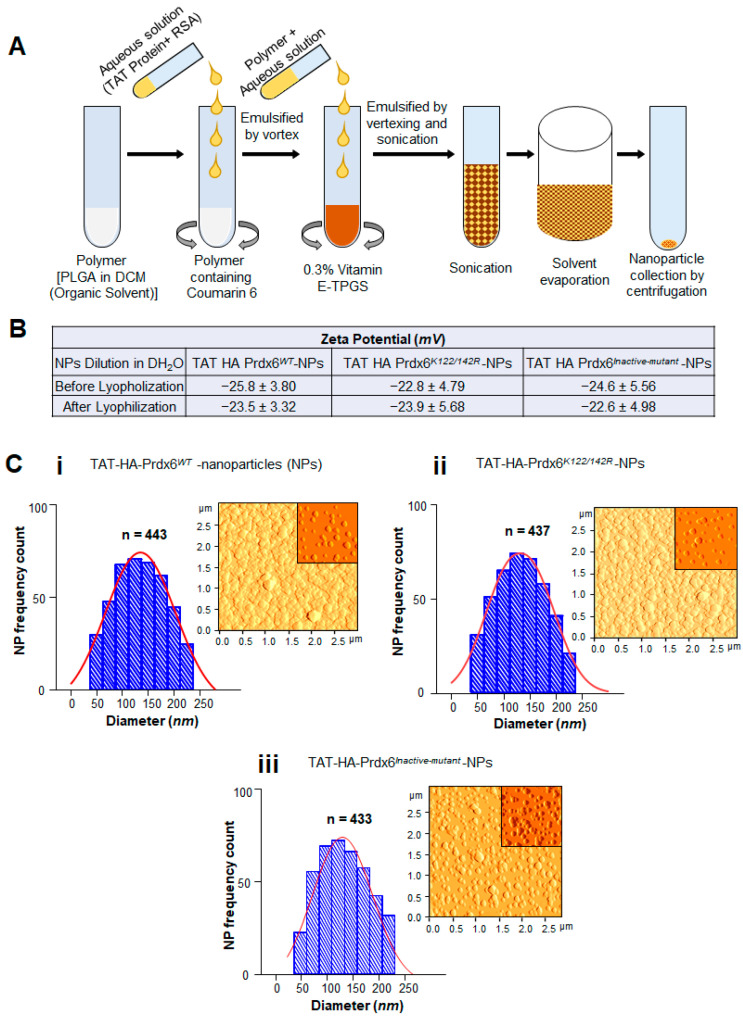
Synthesis and characterization of TAT-HA-Prdx6*^WT^*, TAT-HA-Prdx6*^K122/142R^*, and TAT-HA-Prdx6*^Inactive-mutant^* protein-loaded nanoparticles. (**A**) Schematic representation of TAT-HA-Prdx6 protein analog-loaded nanoparticles made by the emulsion solvent evaporation technique [61,74]. (**B**) Zeta potential of TAT-HA-Prdx6*^WT^*-NPs, TAT-HA-Prdx6*^K122/142R^*-NPs, and TAT-HA-Prdx6*^Inactive-mutant^*-NPs before and after lyophilization. Surface charge (zeta potential) of encapsulants prepared by mixing 1:50 ratio of TAT-HA-Prdx6 protein analog-NPs with DH_2_O. Standard error (mean ± SD) for *n* = 3. (**C**) Representation of atomic force microscopy (AFM) images showing homogenous particle distribution. AFM image (inset, image after dilution) and corresponding size (diameter—nm) distributions of TAT-HA-Prdx6 protein analog NPs.

As noted in Materials and Methods, next, we prepared TAT-HA-Prdx6 analog-loaded PLGA-NPs. TAT-HA-Prdx6*^WT^*-NPs, TAT-HA-Prdx6*^K122/142R^*-NPs, and TAT-HA-Prdx6*^Inactive-mutant^* -NPs were stabilized with Vitamin E-TPGS using the double emulsion solvent evaporation method, as shown in Figure 2A. An aqueous solution containing 10 mg of each of Prdx6 analog along with a stabilizer, RSA (rat serum albumin) and PLGA in DCM with/without Coumarin-6 were used to generate NPs. We found that 100 mg of PLGA and a concentration of 0.3% Vitamin E-TPGS (*w/v*) were the best for nanoparticles formulation [61,74,87]. The characterization of NPs using DLS and AFM analyses showed that TAT-HA-Prdx6 analog-NPs were spherical and submicron size of 50 nm to 250 nm with a negative zeta-potential of ≈23 (Figure 2B). The mean hydrodynamic diameter of the TAT-HA-Prdx6 analog-NPs was greater than the AFM diameter with negative zeta potential as assessed by DLS. However, analysis revealed no significant difference in the zeta potential of TAT-HA-Prdx6 analog-NPs measured before and after lyophilization (Figure 2B), emphasizing that TAT-HA-Prdx6 analog-NPs concentration can be adjusted for delivery.

### 3.3. In Vitro Release, Stability, and Activity of TAT-HA-Prdx6 Analog-Loaded PLGA-NPs and Encapsulation Efficiency

To examine the TAT-HA-Prdx6 protein release from TAT-HA-Prdx6 analog-NPs, we first selected a physiologically relevant buffer that should not denature TAT-HA-Prdx6 protein identity/integrity during in vitro release study. Moreover, the normal tear fluid pH range is about 7 to 7.25 [80,81] and the aqueous humor of the human eye pH range from 7.32 to 7.60 has been reported in various studies. Since the overall pH of the eye fluid is neutral, ranging from 7 to 7.4 [88], we used phosphate-buffered saline (PBS, pH 7.3) containing 0.025% sodium azide for in vitro release assay. The quantitation of released protein showed the following. (1) TAT-HA-Prdx6*^Inactive-mutant^* protein released from TAT-HA-Prdx6*^Inactive-mutant^*-NPs in PBS buffer was 6.2 ± 1.05% (day (d): 1), 23.6 ± 1.92% (day: 5), 46.5 ± 4.23% (day: 10), 71.9 ± 5.34% (day: 15); and 99.09% (day: 21). (2) Total released TAT-HA-Prdx6*^WT^* protein from TAT-HA-Prdx6*^WT^*-NPs was 6.74 ± 0.71% (day: 1), 26.4 ± 2.04% (day: 5), 49.5 ± 3.93% (day: 10), 73.7 ± 6.16% (day: 15), and 99.90% (day: 21). (3) TAT-HA-Prdx6*^K122/142R^* protein released from TAT-HA-Prdx6*^K122/142R^* -NPs was 6.9 ± 1.31% (day: 1), 28 ± 2.09% (day: 5), 52.2 ± 5.89% (day: 10), 75.3 ± 6.78% (day: 15) and 98.93% (day: 21) TAT-HA-Prdx6*^K122/142R^* protein released from TAT-HA-Prdx6*^K122/142R^*-NPs. Taken together, our data revealed that the TAT linked-Prdx6 protein release profiles from all three kinds of NPs were almost similar, and the average release of all three Prdx6 analog was ≈99% as observed on day 21.

**Figure 3:**
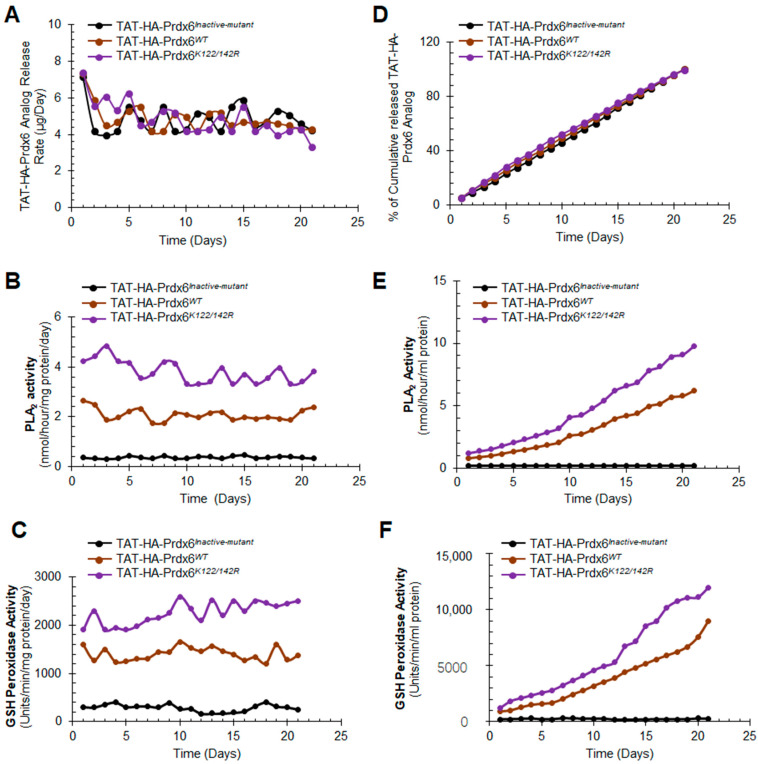
Stability and activity of the TAT-HA-Prdx6*^WT^*, TAT-HA-Prdx6*^K122/142R^*, and TAT-HA-Prdx6*^Inactive-mutant^* protein released from NPs (without Coumarin-6) in PBS (pH 7.3). (**A**,**D**) Release pattern of TAT-HA-Prdx6 analog from PLGA-NPs in PBS (pH 7.3) at 37 °C using sandwich ELISA method. Equal volume of released protein (**A**: Protein released collected at every 24 h and **D**: Cumulative release of protein collected from 0 to 21 days) were used for the sandwich ELISA assay to measure the availability of the released TAT-HA-Prdx6 analog. The data represent the mean ± SD from three independent experiments. (**B**,**C**,**E**,**F**) Prdx6 activity of TAT-HA-Prdx6^WT^ or its mutant proteins released from NPs in PBS. Equal volume of released TAT-Prdx6 protein were processed to measure (**B**,**E**) acidic calcium-independent phospholipase A2 (aiPLA_2_) and (**C**,**F**) glutathione (GSH) peroxidase activity following the company’s protocols and described in our published reports [10].

To assess the encapsulation efficiency and amount of released TAT-HA-Prdx6 analog, a standard plot for different concentrations of TAT-HA-Prdx6 (0.156 to 10 µg/mL) was plotted. The plot showed a linear calibration curve for known TAT-HA-Prdx6 concentrations ranging from 0.156 to 10 µg/mL (*R*^2^ = 0.8527). By calculating the total amount of TAT-HA-Prdx6 protein that was used for loading to PLGA and the amount that could not be entrapped, we quantified the loading efficiency of TAT-HA-Prdx6*^Inactive-mutant^*, TAT-HA-Prdx6*^WT^*, and TAT-HA-Prdx6*^K122/142R^* from the NPs, and these were 60 ± 4.2% (*n* = 3), 69 ± 5.3% (*n* = 3), and 65 ± 3.1% (*n* = 3), respectively. Moreover, the data analysis showed that the TAT-HA-Prdx6 analog was well-retained and stable up to the observation period of 21 days or beyond in the PBS buffer containing RSA (rat serum albumin) and sodium azide as stabilizing and preserving reagents (Figure 3).

Prdx6 exerts its protective action through aiPLA_2_ and GSH peroxidase activities [10]. Hence, we intended to examine whether these activities of released TAT-HA-Prdx6 analog were retained during the process of encapsulation. Toward this, the released protein was collected and processed to examine aiPLA_2_ (Figure 3B,E) and glutathione (GSH) peroxidase (Figure 3C,F) activities. We observed that TAT-HA-Prdx6*^WT^* and TAT-HA-Prdx6*^K122/142R^* proteins maintained their enzymatic activities responsible for cytoprotection. As expected, we found that Sumoylation-deficient TAT-HA-Prdx6*^K122/142R^* could have higher GSH peroxidase and aiPLA_2_ activities compared to wild-type TAT-HA-Prdx6 (Figure 3), as reported previously [10].

### 3.4. Cellular Uptake and Cytoprotective Effects of TAT-HA-Prdx6 Analog-Loaded PLGA-NPs in hLECs In Vitro

**Figure 4:**
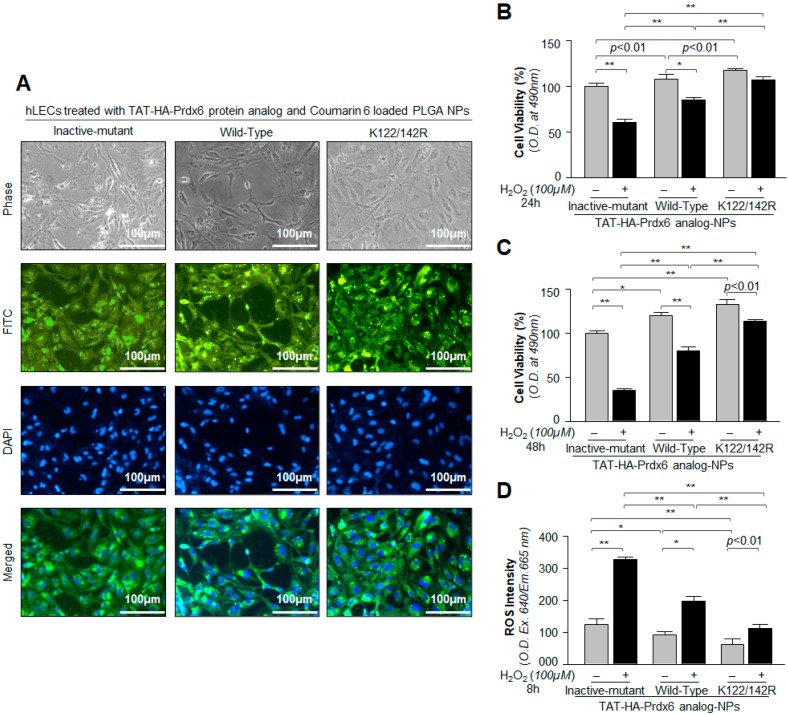
Analysis of Prdx6 analog-loaded PLGA-NPs cellular internalization and evaluation of cytoprotective potential against H_2_O_2_-induced oxidative cell death. (**A**) A representative of photomicrographs displaying the internalization of Prdx6 analog-loaded PLGA-NPs (with Coumarin-6, a fluorescence marker) in hLECs as indicated. (**B**–**D**) hLECs treated with Sumoylation-deficient protein TAT-HA-Prdx6*^K122/142R^*-NPs engendered higher resistance against oxidative stress than TAT-HA-Prdx6*^WT^*-NPs. hLECs were incubated with TAT-HA-Prdx6*^WT^* and its mutant proteins loaded NPs for 6h as indicated; then, the cells were tryspinized and harvested on coverslips to take photomicrographs or in 96-well plates for ROS and MTS analyses. Then, 24 h later, hLECs were exposed to 100 µM of H_2_O_2_, as indicated in figure. After 8 h, ROS intensity was measured with CellROX deep red reagent (**D**), and 24 h and 48 h later, cell viability was analyzed by MTS assay (**B**,**C**). Histogram values represent the mean ± SD from three independent experiments. TAT-HA-Prdx6*^Inactive-mutant^*-NPs vs. TAT-HA-Prdx6*^WT^*-NPs and TAT-HA-Prdx6*^K122/142R^*-NPs; TAT-HA-Prdx6*^WT^*-NPs vs. TAT-HA-Prdx6*^K122/142R^*-NPs; * *p* < 0.05 and ** *p* < 0.001.

The therapeutic effect(s) of drug- or protein-loaded-NPs were heavily attributed to NPs cellular internalization, sustained retention, and biocompatibility [89]. Hence, to examine whether the formulated TAT-HA-Prdx6 analog could internalize in cells, we observed that TAT-HA-Prdx6 analog-NPs could internalize into cells exposed to Prdx6 analogs-NPs containing C6, a fluorescent dye. Figure 4A showed representative photomicrographic images of cells exposed to TAT-HA-Prdx6 analog-NPs containing C6. The images were captured using florescence microscope fitted with a camera (Nikon, Eclipse Ti) through phase channel (gray), FITC channel (green), DAPI channel (blue), or a merge of FITC and DAPI (green and blue).

Next, we examined the protective potential of TAT-HA-Prdx6 analog-NPs. To this end, hLECs pretreated with the Prdx6 analog-NPs suspension were exposed to H_2_O_2_-induced stress, and the viability of cells was assessed using MTS assay at different time intervals, as indicated in the figure. MTS assay at 24 h (Figure 4B) and 48 h (Figure 4C) revealed that cells treated with TAT-HA-Prdx6*^WT^*-NPs had significantly higher resistance and had better survival against H_2_O_2_-induced oxidative stress than TAT-HA-Prdx6*^Inactive-mutant^*-NPs, which is a control vehicle. Interestingly, as expected [10], Sumoylation-deficient TAT-HA-Prdx6*^K122/142R^*-NPs treated cells had significantly increased resistance against H_2_O_2_-induced oxidative damage compared to TAT-HA-Prdx6*^WT^*-NPs, demonstrating that Sumoylation-deficient Prdx6-NPs bear a greater protective potential as reported previously [9,10,11,39]. Since Prdx6 provides cytoprotection by removing ROS, next, we quantified the levels of intracellular ROS in TAT-HA-Prdx6 analog-NPs treated cells exposed to H_2_O_2_ stress. The quantitation of ROS by CellRox Deep Red dye showed that Sumoylation-deficient TAT-HA-Prdx6*^K122/142R^*-NPs were highly efficient in removing ROS in comparison to TAT-HA-Prdx6*^WT^*-NPs (Figure 4D). Conversely, GSH-peroxidase site- and PLA_2_ site-deficient TAT-HA-Prdx6*^Inactive-mutant^*-NPs failed to reduce ROS levels (Figure 4D). As a whole, data revealed that TAT-HA-Prdx6 analog was released from NPs and provided cytoprotection and protected cells by reducing cellular oxidative load, thereby demonstrating that Sumoylation-deficient Prdx6-loaded NPs had a better protective efficacy for therapeutic intervention.

### 3.5. Internalization and Protective Potential of TAT-HA-Prdx6 Analog-NPs in Prdx6^−/−^-Deficient Mouse LECs Facing Oxidative Stress

**Figure 5:**
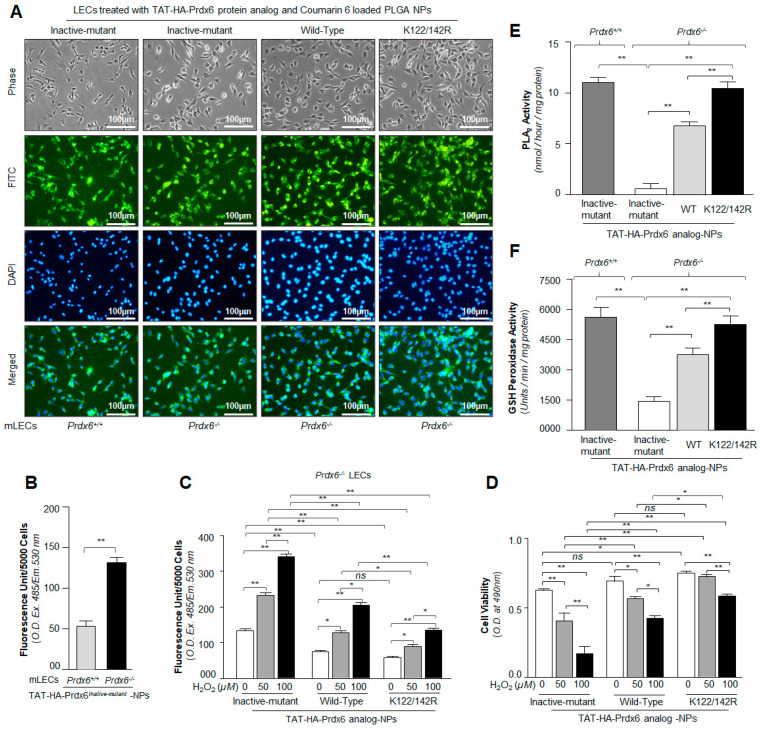
(**A**) Photomicrographs showing cellular internalization of TAT-HA-Prdx6 protein analog-loaded NPs along with Coumarin-6 into *Prdx6^+/+^* mLECs and redox-active (a model for aging) *Prdx6^−/−^* mLECs. *Prdx6^+/+^* and *Prdx6^−/−^* mLECs were incubated with TAT-HA-Prdx6 analog-NPs with Coumarin-6 for 6 h; then, cells were tryspinized and cultured on glass coverslips for taking photomicrograph. Fluorescence and DAPI staining images of cells were recorded after 24 h under inverted fluorescence microscope (Nikon Eclipse Ti-U) as shown. (**B**) Prdx6-deficinet (*Prdx6^−/−^*) LECs displayed significantly increased levels of ROS compared to *Prdx6^+/+^* incubated with TAT-HA-Prdx6*^Inactive-mutant^* -NPs. Histogram values represents the mean ± SD from three independent experiments. *Prdx6^+/+^* vs. *Prdx6^−/−^,* (** *p* < 0.001). (**C**,**D**) Increased protective activity of Sumoylation-deficient TAT-HA-Prdx6*^K122/142R^*-NPs compared to TAT-HA-Prdx6*^WT^*-NPs. Prdx6 analog-NPs-treated *Prdx6^−/−^* LECs were exposed to different concentrations of H_2_O_2_ as indicated in the figure. After 8 h, ROS levels were assessed with H2-DCF-DA dye assay (**C**), and viability of cells was assessed by MTS assay at 48 h of H_2_O_2_ exposure (**D**). Histogram values represent the mean ± SD from three independent experiments. TAT-HA-Prdx6*^Inactive-mutant^*-NPs vs. TAT-HA-Prdx6^WT^-NPs and TAT-HA-Prdx6*^K122/142R^*-NPs; TAT-HA-Prdx6*^WT^*-NPs vs. TAT-HA-Prdx6*^K122/142R^*-NPs (* *p* < 0.05, ** *p* < 0.001). (**E**,**F**) Mutation at Sumoylation sites, K122/142R in Prdx6 augmented aiPLA_2_ and GSH peroxidase activities. mLECs were treated with Prdx6 analog-NPs as shown. Then, 48 h later, a total cell lysate of each sample having equal amounts of protein was prepared and processed for PLA_2_ and GSH peroxidase activities by PLA_2_ and GSH peroxidase assay kits (Invitrogen). Prdx6-deficient LECs showed significantly low levels of PLA_2_ as well as GSH peroxidase activities compared to WT-*Prdx6^+/+^* (**E**,**F**, dark gray bar). TAT-HA-Prdx6^WT^-NPs (**E**,**F**, light gray bars) and Sumoylation-deficient TAT-HA-Prdx6^K122/142R^-NPs (black bar) showed a significant increase in PLA_2_ and GSH peroxidase activities compared to TAT-HA-Prdx6*^Inactive-mutant^*-NPs (open bar) in *Prdx6^−/−^* LECs. Histogram values represent the mean ± SD from three independent experiments. TAT-HA-Prdx6*^Inactive-mutant^*-NPs vs. TAT-HA-Prdx6*^WT^*-NPs and TAT-HA-Prdx6*^K122/142R^*-NPs; TAT-HA-Prdx6^WT^-NPs vs. TAT-HA-Prdx6*^K122/142R^*-NPs (** *p* < 0.001).

With advancing age, the progressive increase in oxidative load-dependent cellular injuries is found to be a major cause for the etiopathologies of many diseases [3,9,11,39,40,82,90]. Thus, we sought to determine whether TAT-HA-Prdx6 analog-NPs were equally effective (as observed in hLECs) in protecting redox-active Prdx6-deficient mLECs [3,4,9,11,14,15,39]. In addition, in the above experiments, we have used hLECs already containing naturally occurring Prdx6; hence, the absolute cytoprotective contribution of exogenously supplied Prdx6 in the form of TAT-HA-Prdx6 analog-NPs was not clear. Thus, to determine the contribution of exogenously supplied TAT-HA-Prdx6 analog via NPs, we utilize Prdx6-deficient (*Prdx6^−/−^*) mLECs [3,4,10,11,15,28,39]. In this experimentation, *Prdx6^+/+^* mLECs were treated with TAT-HA-Prdx6*^Inactive-mutant^*-NPs suspension as a control to examine the protective contribution of endogenous Prdx6, while *Prdx6^−/−^* mLECs were treated with the suspension of the TAT-HA-Prdx6*^Inactive-mutant^*-NPs, TAT-HA-Prdx6*^WT^*-NPs, and Sumoylation-deficient TAT-HA-Prdx6-NPs. The images shown are representative of experiments; Figure 5A showed the internalization of the TAT-HA-Prdx6 analog-NPs containing C6 into *Prdx6^+/+^* and *Prdx6^−/−^* mLECs. Similarly, *Prdx6^+/+^* and *Prdx6^−/−^* mLECs were treated with the NPs (without Coumarin-6) suspension of the TAT-HA-Prdx6*^Inactive-mutant^*-NPs, TAT-HA-Prdx6*^WT^*-NPs, and TAT-HA-Prdx6*^K122/142R^*-NPs to assess their protective efficiency. We observed that TAT-HA-Prdx6*^Inactive-mutant^*-NPs treated *Prdx6^−/−^* mLECs had ≈2 to 3-fold higher ROS load than *Prdx6^+/+^* mLECs (Figure 5B). In contrast, TAT-HA-Prdx6*^WT^*-NPs and TAT-HA-Prdx6*^K122/142R^*-NPs-treated Prdx6-deficient (*Prdx6^−/−^*) mLECs had significantly reduced ROS levels (Figure 5C) with increased cell viability (Figure 5D) in response to H_2_O_2_-induced oxidative stress, suggesting that the formulated NPs were effective at reducing ROS load and enhancing the cell viability of redox-active cells, *Prdx6^−/−^* mLECs. Additionally, the results revealed that Sumoylation-deficient TAT-HA-Prdx6*^K122/142R^*-NPs had a greater cytoprotective potential (Figure 5D), and they did so by lowering the ROS levels (Figure 5C) in comparison to TAT-HA-Prdx6*^WT^*-NPs. In addition, we observed that the increased protective activity was related to a significant increase in the aiPLA_2_ (Figure 5E) and GSH peroxidase (Figure 5F) activities of TAT-HA-Prdx6*^K122/142R^*-NPs compared to TAT-HA-Prdx6*^WT^*-NPs. Taken together, our data revealed that all TAT-HA-Prdx6 analog-NPs successfully internalized into the LECs and protected the cells against the H_2_O_2_-induced oxidative stress being stable and biologically active.

### 3.6. Subconjuctival Administration of TAT-HA-Prdx6 Analog-NPs Prevented Lens Opacity and Delayed the Progression of Cataract Formation in SCRs

**Figure 6:**
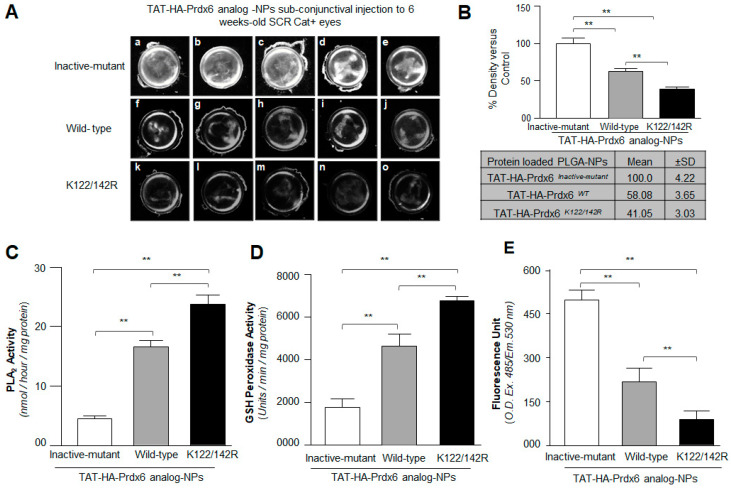
(**A**,**B**) Preventive effect of TAT-HA-Prdx6*^WT^*-NPs and Sumoylation-deficient Prdx6-NPs in the eye on progression of cataract in SCR rats. Six-week-old SCRs were administered subconjuctivally with either TAT-HA-Prdx6*^WT^*-NPs or Sumoylation-deficient TAT-HA-Prdx6*^K122/142R^*-NPs (right eye: 25 µg/10 µL in physiological saline) and TAT-HA-Prdx6*^Inactive-mutant^*-NPs (left eye; 25 µg/10 µL in physiological saline). At the end of the experiment, lenses were removed and photographed using a stereomicroscope; (**A**) Representative photographs showing the opacity of lenses treated with TAT-HA-Prdx6-analog-loaded NPs, as shown. (**B**) The relative density of lenses was measured, and values were presented as histograms. (**C**,**D**) Sumoylation-deficient TAT-HA-Prdx6*^K122/142R^*-NPs had increased aiPLA_2_ and GSH peroxidase activities compared to TAT-HA-Prdx6*^WT^*-NPs. Total protein was isolated from the lenses, and assays were performed for (**C**) aiPLA_2_ and (**D**) GSH peroxidase activities. Histogram values represent the mean ± SD from three independent experiments. TAT-HA-Prdx6*^Inactive-mutant^*-NPs vs. TAT-HA-Prdx6*^WT^*-NPs and TAT-HA-Prdx6*^K122/142R^*-NPs; TAT-HA-Prdx6*^WT^*-NPs vs. TAT-HA-Prdx6*^K122/142R^*-NPs (** *p* < 0.001). (**E**) Reduced ROS generation was observed in Sumoylation-deficient Prdx6 NPs in comparison with Prdx6 WT-NPs. Six-week-old SCRs were administered subconjuctivally with either TAT-HA-Prdx6*^WT^*–NPs or Sumoylation-deficient TAT-HA-Prdx6*^K122/142R^*-NPs (right eye: 25 µg/10 µL in physiological saline) and TAT-HA-Prdx6*^Inactive-mutant^*–NPs (left eye; 25 µg/10 µL in physiological saline). At the end of the experiment, lenses were removed, and homogenates were prepared. ROS levels were monitored with H2-DCF-DA dye assay. Data represent means ± S.D. of three independent experiments. ** p < 0.001.

While in vitro studies conducted would be very useful for documenting the characterization and cytoprotective potential of formulated Prdx6-NPs, we acknowledge that the results obtained from the in vitro studies could only be considered if they could be reproduced in vivo. Considering the wide range of protective and survival activities of Prdx6 [3,4,8,9,14,15,34,39,91,92,93,94,95,96,97], we chose to use the SCR (a model for cataract), which shows a spectrum of biochemical and morphological changes, including oxidative-induced cell biological changes during the progression of cataract [14,71,98,99,100]. Since the SCRs generally develop cataracts at the 9th week, we used 6-week-old rats, administering TAT-HA-Prdx6 analog-NPs by the subconjuctival route. The rats were observed (every third day) for the onset of cataract. Twelve SCRs were used for the study; the number was based on the work of Plaisant et al. [94] and Kubo et al. [14,101]. The right eye of each rat was selected to deliver TAT-HA-Prdx6*^WT^*-NPs or Sumoylation-deficient TAT-HA-Prdx6*^K122/142R^*-NPs (10 µL NPs containing 25 µg of protein of each suspended in physiological saline), whereas the left eye received TAT-HA-Prdx6*^Inactive-mutant^* suspended in physiological saline as control vehicle. At week 9, lenses were isolated from rat eyes and photographed. Figure 6 showed that the lenses from the eyes that had received TAT-HA-Prdx6^WT^-NPs (Figure 6(Af–Aj)) or TAT-HA-Prdx6*^K122/142R^*-NPs (k-o) injection were observed to be clearer (Figure 6A) than the lenses of the eyes that received GSH peroxidase site- and PLA_2_ site-mutant protein, TAT-HA-Prdx6*^Inactive-mutant^* (Figure 6(Aa–Ae)). It was intriguing to observe that NPs containing Sumoylation-deficient Prdx6 protein could have a better protective potential (lower panel, ≈60% reduction in lens opacity vs. control) to preventing cataract formation compared to NPs having Prdx6*^WT^* (middle panel, ≈40% reduction in lens opacity vs. control), demonstrating that Sumoylation-deficient Prdx6*^K122/142R^* could have a greater therapeutic potential. The changes in the transparency of lenses were measured by performing densitometric analysis and values were presented as histograms (Figure 6B).

Next, we wished to examine the status of Prdx6 activities in rat lenses that received TAT-HA-Prdx6-protein analog-NPs subconjuctivally. We examined the aiPLA_2_ and GSH peroxidase activities of Prdx6 of rat lenses after isolation as described previously [10] in the Materials and Methods. Total protein isolated from the TAT-HA-Prdx6 analog-NPs delivered lenses of SCRs were used for the assay. We found that Sumoylation-deficient TAT-HA-Prdx6*^K122/142R^*-NPs administered rat lenses displayed significantly increased aiPLA_2_ and GSH peroxidase activities compared to other two Prdx6 analogs, as shown in Figure 6C,D. As expected, we observed significantly more aiPLA_2_ and GSH peroxidase activities in TAT-HA-Prdx6*^WT^*-NPs or Sumoylation-deficient Prdx6-NPs injected rat lenses than TAT-HA-Prdx6*^Inactive-mutant^*-NPs, indicating a plausible contribution C47 and catalytic triad S32-H26-D140 for GSH peroxidase and aiPLA_2_ activities. Next, we wished to examine the ROS status in these lenses. Whole lenses were homogenized and levels of ROS were assessed [83]. We observed ≈ 60% reduction in the levels of ROS in TAT-HA-Prdx6*^WT^*-NPs compared to TAT-HA-Prdx6*^Inactive-mutant^*-NPs-injected rat lenses. Interestingly, we observed a significantly reduced ROS levels in Sumoylation-deficient TAT-HA-Prdx6*^K122/142R^*-NPs administered rat lenses in comparison with TAT-HA-Prdx6*^WT^*-NPs injected rat lenses. Taken together, our data revealed that TAT- HA-Prdx6 analogs-NPs released biologically active TAT-HA-Prdx6 analog, and subconjuctival injection of Sumoylation-deficient Prdx6-NPs could efficiently delay the lens opacity by normalizing the ROS levels.

## 4. Discussion

A general characteristic of oxidative- or aging-related diseases is a progressive loss of redox homeostasis due to the deterioration of cellular antioxidant defense response. Under normal physiological conditions, the levels of ROS are finely and tightly regulated by the cellular antioxidant defense system [3,10,73,82,102,103]. Conversely, the amplification of ROS due to antioxidant dysregulation during aging or oxidative stress leads to pathological signaling, such as oxidative nucleic acid and protein damage [8,9,11,82,104,105]. In addition to the endogenous production of ROS, environmental stressors, such as light, radiations, chemicals, and xenobiotic also accelerate ROS-driven oxidative load [8,23,52,82,106,107,108]. Thus, both intrinsic (reduced capacity of antioxidants) and extrinsic factors play a critical role in the progression of aging/oxidative stress-associated diseases. The eye is highly exposed to light and is a target for various kinds of environmental pollutants. To cope with these stressors, the eye lens has developed an antioxidant defense system and is enriched with antioxidant enzymes [10,14,82]. In our previous studies, we have shown that lens cells or lenses treated with multifunctional antioxidant protein Prdx6 or its protective mutant, Prdx6*^K122/142R^*-linked to TAT (transactivation of transcription) transduction domain engender resistance against environmental stressors [3,4,8,9,10,11,14,23,39,52,82,83,93,108]. In addition, we have reported that the subconjuctival injection of TAT-HA-Prdx6 protein delays/prevents lens opacity in Shumiya cataract rat (SCR) [14]. In this study, our goal was to further augment the protective potential of TAT-linked Sumoylation-deficient Prdx6*^K122/142R^* [10] by using nanoformulation technology to develop it as a therapeutic molecule to prevent blinding diseases and other oxidative stress-related diseases.

Prdx6 is a naturally occurring endogenous protein with multiple functionalities responsible to control cellular homeostasis [3,4,8,10,11,12,13,14,52,82]. We surmised that the nanoparticle formulation of endogenous protective proteins such as Prdx6 should have advantages, because it might be cytocompatible when released from nanoparticles within the cellular microenvironment. With this notion, we prepared TAT (protein transduction domain)-linked wild-type Prdx6 or Sumoylation-deficient Prdx6-loaded PLGA nanoparticles (NPs) and tested their potential in rescuing LECs against oxidative stress in vitro and also assessed its preventive effect on the onset of SCR cataractogenesis when injected subconjunctivally. As shown in Figure 4, Figure 5 and Figure 6, Prdx6 analog-loaded PLGA-NPs were cytocompatible with human and mouse LECs as well as SCR LECs/lenses. We observed that Sumoylation-deficient Prdx6 could have greater protective potential (Figure 4 and Figure 5), and the delivery of Sumoylation-deficient Prdx6-NPs delayed the SCR lens opacity (Figure 6) compared to wild-type TAT-HA-Prdx6-NPs. Moreover, the TAT domain has 11 amino acids (aa; YGRKKRRQRRR), the TAT-linked protein can internalize in cells with 100% capacity, and also, TAT proteins can across the plasma membrane as well as the blood–brain barrier [9,10,11,14,53,56,109]. In addition, based on previous reports showing that the TAT-linked proteins can have transcellular behavior [110,111], we believe that cell-to-cell movement and the internalization of TAT-liked Prdx6 or Sumoylation-deficient Prdx6 can be more efficient to provide protection due to cellular availability within the cellular microenvironment. However, at present, we do not know if TAT-linked Prdx6 is released from cells and internalized into adjacent cells in vitro or in vivo and requires further investigation. Moreover, we have reported that Prdx6 is Sumoylated at K122 and K142 residues by Sumo1, and aberrant Sumoylation of Prdx6 in response to oxidative stress and aging leads to its degradation and reduced protective activity [9,10]. Oxidative stress-evoked erratic Sumoylation of nuclear and cytoplasmic proteins alters their genetically destined functions and cellular status [9,10,46,112,113,114]. Notably, we have reported that Sumoylation-deficient TAT-HA-Prdx6 bears enhanced aiPLA_2_ and GSH peroxidase activities of Prdx6 and provides significantly increased cytoprotection by escaping ROS-driven aberrant Sumoylation-mediated adverse signaling [10]. Interestingly, we observed that Sumoylation-deficient Prdx6-NPs could have a better protective potential in removing ROS load (Figure 4 and Figure 5).

Progress in translating research findings to treatments of oxidative/aging-linked blinding pathobiology has been limited due to the non-availability of suitable model(s) and ideal drug/protein delivery systems. However, aging and oxidative stress have common denominators; thus, the oxidative- and age-related pathologies share a series of interlinked pathogenesis, and studies reveal that delaying or treating one oxidative/aging disease can withstand other diseases [115,116,117]. Blinding diseases such as cataract, glaucoma, and macular degeneration have been shown to be linked to oxidative stress and aging, and the alleviation of pathogenesis linked to these diseases requires prolonged treatment. In the present study, we have used SCR as a model to test the protective potential of Prdx6 analog-NPs. Although the etiology of cataract formation in SCR is related to mutations in the lanosterol synthase gene, we and other investigators have shown that in the SCR, the onset of cataractogenesis involves the reduced expression of Prdx6 and a spectrum of biochemical and morphological changes. Notably, the features of these abnormal changes are similar to those found in cataractogenesis evoked through oxidative stress and growth factors such as TGFβs [3,14,98,99]. In this work, we found that indeed, TAT-HA-Prdx6^WT^ or Sumoylation-deficient Prdx6*^K122/142R^*-NPs when administered subconjuctivally, sustained Prdx6 release, prevented lens opacity, and protected LECs (Figure 6). PLGA is an FDA-approved product and is biodegradable with the sustained release of encapsulated protein/drug to the target with minimum toxicity. Thus, we think that the formulation can be used for preclinical or clinical studies [66,118,119,120].

Ophthalmic drug solutions are preferred within a physiological pH range [66]. PBS (pH 7.3–7.4) and stimulating tear fluid (STF, pH 7.2) near physiological pH have been previously used for in vitro release assays as well as for the administration of drugs in the eye [80,81,88,121,122]. Based on these reports, we performed our studies and validated the quantity, stability, and activities of in vitro released TAT-HA-Prdx6 analog (from NPs) by using Sandwich ELISA and aiPLA_2_ and GSH peroxidase activities assays (Figure 3) [10,39]. Previously, using SOD enzyme activity assay, the release of a stable and active form of SOD over 7 days in PBS was demonstrated for SOD-loaded PLGA-NPs [61]. Furthermore, for the ocular drugs/protein/DNA-NPs delivery, NPs size is critical, since particle retention in the subconjunctival location depends on size. Particles larger than 10 µm have been found to be irritable and uncomfortable. In our work, nanoparticles were about 250 nm with negative (approximately −22 to −26 mV) zeta potential, suggesting their suitability for subconjunctival delivery. Importantly, our present data reveal that TAT-HA-Prdx6*^K122/142R^*-NPs with increased enzymatic activity and stability, efficiently protected LECs against oxidative stress and delayed the progression of cataract in SCRs by limiting ROS-driven damage.

## 5. Conclusions

**Figure 7:**
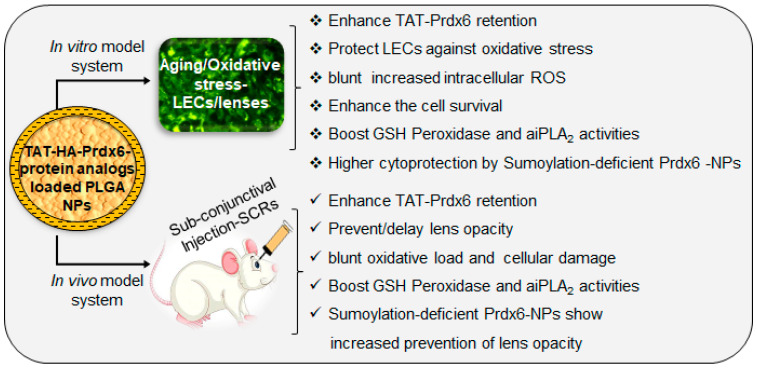
Diagrammatic illustration of the Sumoylation-deficient Prdx6 mutant loaded NPs delivery and protective efficiency in vitro and in vivo.

In summary, we have shown for the first time that Sumoylation-deficient Prdx6-NPs are highly efficient to mitigate the effect of oxidative stress-induced cellular damage. Interestingly, subconjunctival administration of the NPs in the SCR eyes delays cataract formation by reducing ROS-mediated insults. We demonstrated that the Sumoylation-deficient Prdx6-NPs were cytocompatibile and biologically active, having significantly increased GSH peroxidase and aiPLA_2_ activities, and they provided significantly better protection and prevention. While it appears that Sumoylation-deficient Prdx6-NPs-driven protection and delay in cataract formation is primarily dependent upon reduction in ROS overshooting-mediated tissues damage, we acknowledge that other protective mechanism(s) may be involved, which warrant further investigation; the investigation of such signaling and unraveling the mechanisms of Prdx6-mediated blocking of the adverse signaling are future research directions of our laboratory. However, our findings demonstrate the benefit of Sumoylation-deficient Prdx6-NPs in delaying or preventing cataractogenesis in SCR and in blocking oxidative stress-driven adverse signaling. We propose the use of Sumoylation-deficient Prdx6 in the form of NPs as a novel therapeutic molecule to treat or delay oxidative-stress-driven diseases, including cataractogenesis (Figure 7).

## Data Availability

Data are contained within the article.
